# Should continuous deep sedation until death be legally regulated in Switzerland? An exploratory study with palliative care physicians

**DOI:** 10.1177/26323524231219509

**Published:** 2023-12-25

**Authors:** Martyna Tomczyk, Roberto Andorno, Ralf J. Jox

**Affiliations:** Institute of Humanities in Medicine, Lausanne University Hospital and University of Lausanne, Av. de Provence 82, Lausanne CH-1007, Switzerland; Institute of Biomedical Ethics and History of Medicine, University of Zurich, Zurich, Switzerland; Institute of Humanities in Medicine, Lausanne University Hospital and University of Lausanne, Lausanne, Switzerland; Palliative & Supportive Care Service, Chair in Geriatric Palliative Care, Lausanne University Hospital and University of Lausanne, Lausanne, Switzerland

**Keywords:** continuous deep sedation, legal regulation, palliative care, physicians, qualitative study, Switzerland

## Abstract

**Background:**

In Switzerland, continuous deep sedation until death (CDSUD) is not legally regulated and the current clinical practice guidelines on palliative sedation from 2005 do not refer to it. In contrast, in France, a neighbouring country, CDSUD is regulated by a specific law and professional guidelines. International studies show that in culturally polymorphic countries, there are variations in the end-of-life practices between linguistic regions and that a linguistic region shares many cultural characteristics with the neighbouring country.

**Objectives:**

This study aimed to explore the attitudes of palliative care physicians from the French-speaking part of Switzerland on the question of whether CDSUD should be legally regulated in the country, and to identify their arguments. Our study also aimed to assess whether a hypothetical Swiss law on CDSUD should be similar to the current legal regulation of this practice in France.

**Design:**

We conducted a multicentre exploratory qualitative study based on face-to-face interviews with palliative care physicians in the French-speaking part of Switzerland.

**Methods:**

We analysed the interview transcripts using thematic analysis, combining deductive and inductive coding.

**Results:**

Most of the participants were opposed to having specific legal regulation of CDSUD in Switzerland. Their arguments were diverse: some focused on medical and epistemological aspects of CDSUD, whereas others emphasized the legal inconvenience of having such regulation. None had the opinion that, if CDSUD were legally regulated in Switzerland, the regulation should be similar to that in France.

**Conclusion:**

This study allows to better understand why palliative care physicians in French-speaking Switzerland may be reluctant to have legal regulation of CDSUD. Further studies covering the whole country would be needed to gain a more complete picture of Swiss palliative care physicians on this question.

## Introduction

In the context of palliative care, continuous deep sedation until death (CDSUD) is an important therapy of last resort.^1–3^ However, this practice remains controversial at both the clinical and ethical levels, especially due to its irreversibility and the arguable lack of proportionality.^4^

Although there are some international data on the prevalence of CDSUD,^5,6^ (including data gathered in Switzerland^7^), it is impossible, or at least imprudent, to make comparisons and determine whether this practice is ‘rare’, ‘common’ or ‘frequent’, because of the terminological and conceptual inconsistencies, diverse applications and varying perceptions of these practices. Many international comparative studies show that CDSUD is influenced by the culture of the country in which it is practised, and particularly by the legal and social contexts. Several cultural differences in the attitudes of palliative care practitioners and the decisions and practices of CDSUD have also been observed between countries.^8–10^ However, all the data suggest a trend towards an increase in the use of CDSUD across the world. For example, in Switzerland, between 2001 and 2013, this practice increased from 4.7% to 17.5%.^7^

According to linguistic theory, language is an important part of the culture of a country.^11,12^ Some studies show that in culturally polymorphic countries, there are variations in the end-of-life practices between linguistic regions and that a linguistic region shares many cultural characteristics with the neighbouring country of the same language.^13–15^ For instance, Van den Block *et al.*^15^ have demonstrated that, while there is a tendency towards the use of CDSUD in the Walloon region (the French-speaking part of Belgium), euthanasia is more frequently practised in Flanders (the Dutch-speaking part of the country). A study performed by Chambaere *et al.*^14^ shows that, in the metropolitan Brussels-Capital Region, where both language communities are represented, French-speaking physicians perform CDSUD more often than their Dutch-speaking colleagues. The findings suggest that these differences are present irrespective of the geographical separation, but more in line with the language used.

Switzerland offers an opportunity to explore in depth and from various perspectives the cultural-linguistic influence on end-of-life issues, such as CDSUD. The country has four official languages (German, French, Italian and Romansh), with their own linguistic-geographical areas of very different sizes. Previous studies confirm that there are cultural differences in medical end-of-life decisions and practices between the German-, French- and Italian-speaking regions of the country.^16,17^ For example, a study highlights that CDSUD is used more frequently in the Italian-speaking region than in the French- and German-speaking areas.^17^ However, to the best of our knowledge, these findings were not compared with what is practised in the neighbouring countries, such as Italy, France and Germany. Another study, performed by Faeh *et al.*,^18^ although not dealing with end-of-life issues but with risk factors and causes of death across Switzerland, clearly shows that the situation in the French-speaking part of the country is more similar to that in France and that the setting of the German-speaking part of Switzerland corresponds more to that in Germany. This suggests that issues relating to end-of-life care in the French-speaking part of Switzerland, especially CDSUD, may be influenced by the French context.

In this regard, it is interesting to consider that France is the first and only country in the world to have explicitly and precisely regulated CDSUD at the legal level.^19^ The law enacted in February 2016, named the ‘Claeys–Leonetti law’ after its initiators, Alain Claeys and Jean Leonetti, members of parliament, allows CDSUD at the patient’s request in two situations: (1) in the case of a serious and incurable condition endangering life in the short term, in so far as the patient’s suffering is refractory to treatment, and (2) when the decision of a patient with a serious and incurable condition to stop treatment is life-threatening in the short term and is likely to cause unbearable suffering. The law also provides that where the patient is unable to express his or her will and the physician estimates that the life-sustaining treatment constitutes an ‘unreasonable obstinacy’, he or she can decide to forego it and apply CDSUD.^20^ It should be noted that this CDSUD aims directly for the level of unconsciousness (Richmond Agitation Sedation Scale (RASS)-4/-5) and not patient relief. In other terms, the goal is a completely unresponsive patient, even if the patient experiences relief at lesser doses.

In Switzerland, CDSUD is not legally regulated, and in the current national clinical practice guidelines on palliative sedation this practice is not explicitly mentioned.^21^ On the other hand, the new guidance issued by the Central Ethics Commission of the Swiss Academy of Medical Sciences, incorporated into the professional code of healthcare workers, explicitly mentions this treatment; CDSUD may only be performed in dying patients and, contrary to the rules of the ‘Claeys–Leonetti law’,^20^ the depth of sedation applied is to be symptom guided.^22^

The objective of our study was to explore how palliative care physicians working in the French-speaking part of Switzerland perceive a hypothetical legal regulation of CDSUD in the country. Our study also aimed to assess whether in the eyes of the participants such hypothetical legislation on CDSUD should be similar to the law in France. To the best of our knowledge, and at the time of writing this article, no studies have comprehensively investigated these questions.

## Materials and methods

### Study design

Between February and November 2019, we performed a multicentre exploratory qualitative study based on face-to-face interviews with physicians who were working or had previously worked in a specialized palliative care unit in the French-speaking part of Switzerland. The overall study focused on CDSUD as an alternative to assisted suicide in Switzerland. The results of this main study have been published elsewhere.^23^ We conducted two secondary data analyses, one of which, focusing on assistance in suicide, has already been published.^24^ Here, we publish the results of the other secondary data analysis of CDSUD. In this article, we summarize the methodological aspects of the main study (data collection)^23^ and then elaborate on the method of the secondary data analysis concerning the legal aspects of CDSUD in Switzerland. All methodological aspects are detailed in Supplemental File 1 according to the COREQ (COnsolidated criteria for REporting Qualitative research) checklist.^25^

### Data collection

Four palliative care units certified by the Swiss Association for Quality in Palliative Care and located in the French-speaking part of Switzerland served as the basis for sampling. The inclusion process was progressive and the sample size was determined by data saturation.^23^ MT, a postdoctoral researcher in palliative care, with expertise in ethics, law, linguistics and qualitative research, conducted face-to-face interviews with physicians, using the interview guide.

Prior to this study, MT had no relationship with the participants. At the beginning of each interview, the researcher clarified that this study was carried out as part of her postdoctoral project and provided comprehensive research-related information.

### Data analysis

All the interviews were transcribed by MT, who was the only person with access to the recordings and integral transcripts. RA and RJJ had access to anonymized parts of the transcripts. The data were manually analysed by MT; no software tools were used.

The thematic analysis was performed using a hybrid approach of deductive and inductive coding. For the analysis of the transcripts, a deductive approach was followed using a simple framework.^26^ Two major themes were identified beforehand: (1) a hypothetical legal regulation of CDSUD in Switzerland in general, including arguments for and against and (2) the specific legal regulation of CDSUD as it exists in France, and arguments for and against. For this second theme, the researcher explained the French law on CDSUD in detail beforehand, clarifying precisely the three situations in which CDSUD can be requested by patients (see ‘Introduction’ section). These two main themes were researched in all the transcripts. In the next step, minor themes were identified inductively as they emerged from the analysis and were not predefined. After that, transversal analyses of all the transcripts were conducted, and all the themes were synthesized. It is important to note that the analysis was carried out on the original French versions, and only the results and quotations were translated into English. RA and RJJ contributed to the process of data interpretation.

## Results

### Sample size and characteristics

We contacted nine palliative care units in the French-speaking part of Switzerland and received responses from all of them. Of the nine units, six agreed to participate and we included four of them in the study. We conducted interviews with 12 physicians from these four palliative care units, but we were only able to transcribe and include 10 of the interviews in our analysis. We excluded two interviews: one because it was too short and the other because of a technical problem rendering the recording inaudible. The duration of the interviews ranged from 13 to 46 min. Detailed characteristics of the participants can be seen in Table 1.

**Table 1.** Characteristics of the participants.

Number of participants whose interviews were included in the study	*n* = 10
Gender	
Female	*n* = 5
Male	*n* = 5
Age	
Median age	49 years (range 38–61)
Principal medical specialty*	
Internal medicine	*n* = 8
Anaesthesiology	*n* = 1
General practitioner	*n* = 1
Training in palliative care^$^	
Yes	*n* = 7
No	*n* = 3
Experience in palliative care^‡^	
>10 years	*n* = 7
<10 years	*n* = 3
Current workplace	
Palliative care unit	*n* = 7
Other (mobile palliative care team, ambulatory consultations)	*n* = 3^§^

* Palliative care is not a specialty in Switzerland but only a subspecialty.

$ For example: Certificate of Advanced Studies (CAS), Diploma of Advanced Studies (DAS) and/or Master.

‡ Until 2019.

§ Various configurations, depending on the percentage of the work.

### Hypothetical legal regulation of CDSUD in Switzerland

**Figure 1.**
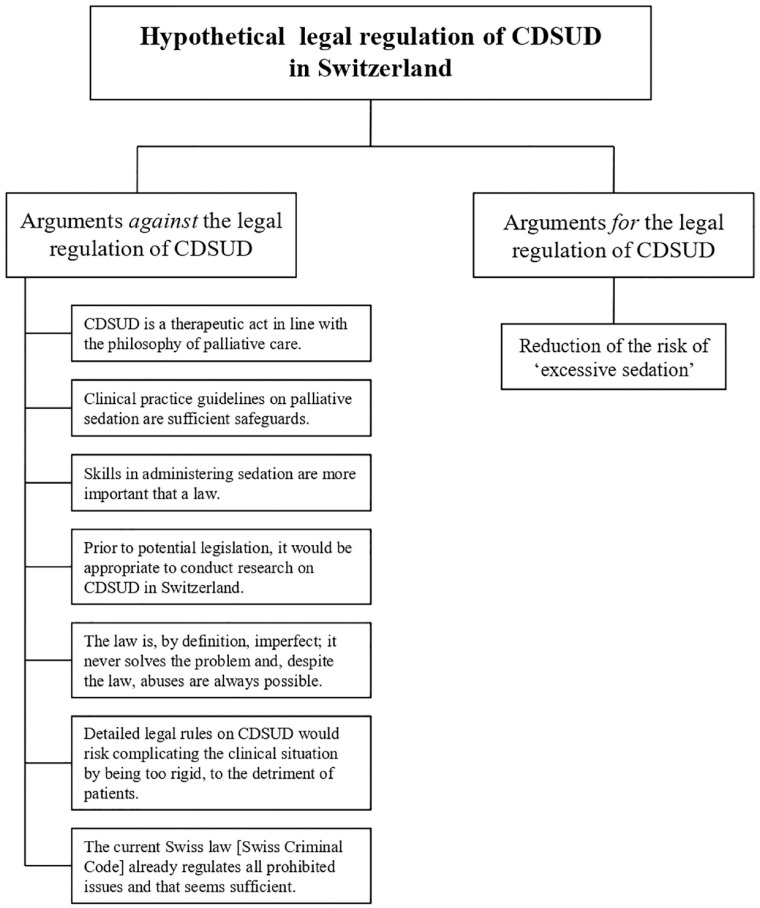
Main results.

Only one participant was fully in favour of the legal regulation of CDSUD. Two participants hesitated, without giving a clear answer: they presented arguments both for and against. Most of the participants were firmly against legally regulating CDSUD in Switzerland. The results presented in this section are schematically summarized in Figure 1. All quotations are presented in Table 2.

**Table 2.** Arguments for and against the legal regulation of CDSUD: Quotations 1–17.

Quotation 1: ‘[. . .] the law would make it possible to regulate that there is no excessive sedation. . . [. . .] Now, each physician can prescribe Midazolam to each patient. . . by agreement. It’s done and *voilà*. . . That is a little tricky’.
Quotation 2: ‘Of course, we must have safeguards, we must prevent people from doing anything and no matter how’.
Quotation 3: ‘So, I think that [CDSUD] should be legislated. Clear criteria are needed to avoid any abuse’.
Quotation 4: ‘I think that it [CDSUD] is a therapeutic act that is up to the doctor. We are not legally wondering whether to introduce an antibiotic or not. That is similar, although the indications are different’.
Quotation 5: ‘[– Should CDSUD be legally regulated?] – No, no! Not at all. For me, this is a palliative care treatment, as long as it is not diverted to something else’.
Quotation 6: ‘Here [in this palliative care unit], we are sufficiently imbued with respect for life. We’re not going to do just anything, anyhow. I don’t need a law that regulates my work more than the one that exists now [. . .]’.
Quotation 7: ‘A law. . . I don’t know. . . There are clinical practice guidelines issued by the Swiss Society for Palliative Care and Medicine. There is also an international consensus. I believe that is enough. I think that in Switzerland, at least in my practice, we don’t need to legislate’.
Quotation 8: ‘[. . .] legislate on sedation. . . I don’t really want to. In contrast, I really want the rules of good practice, which also have legal force, to be respected. We should work in line with the Swiss clinical guidelines [. . .] They are good medical guidelines. They accept the fact that there may be complex, particular situations in which certain aspects of this practice could be modulated’.
Quotation 9: ‘I think that it depends on the expertise of the people [physicians]. It is true that a physician who has never practised sedation, who is not in the field of palliative care, may be tempted to do. . . I don’t know. . . It is certain that sedation is practised in another way in surgery, for example; they [surgeons] may push more morphine. I don’t know. I think that sedation requires some expertise (fine-tuning the dosage, adapting to the indications)’.
Quotation 10: ‘Two or three years ago, the French Society for Support and Palliative Care began [. . .] to create a typology of sedations to try to better understand this practice [. . .]. That [studies concerning types of sedation] has not been performed [in Switzerland]. That might be interesting to have [data], to see how it goes before eventually legislating. . .’.
Quotation 11: ‘I have the impression that the laws seldom prevent abuses and that [they] often complicate things which could be simple’.
Quotation 12: ‘By definition, the law is stupid stuff. It’s supposed to sort things out and each time it just can’t be clear. Otherwise, there wouldn’t be the thousands of lawyers who earn millions a year for finding a distinction between a comma and no comma. So, imagining that the law can regulate things is completely illusory’.
Quotation 13: ‘The only thing I see [in the decision-making process] is the problem of values, but I think it is a much more fundamental problem, typical of all medicine: it is said that patients must have an informed choice and informed consent, and I think in 90% of cases, it’s not a choice. It is advice with my values that I am imposing on or influencing the other to follow, what I think is right. So, for me, the danger is that the doctor will impose his values of good sedation and of a good death to the patient. But I can hardly imagine how legally we could frame this, except with a second opinion, with the principle of collegiality and with a second doctor. But the second doctor will rarely say: “Ah, you influence him, this is bad sedation, we are not going to go.” Even this guardrail is not going to be very effective’.
Quotation 14: ‘There is a legal side and there is a practical side. If I see the practical side, it is already a long time to agree on sedation. Sometimes when we do a debriefing with the healthcare team, we hear “We could have done it a long time ago,” “I don’t understand why we haven’t talked about this before?,” “Why haven’t we done this before? So, it always takes a while.” [. . .] So, sedation is already a long time, even a very long time, to be put in place. If, in addition, at the legal level, there are complications (because if it is framed by law, we can imagine that there is neutral expertise, that there is an expert who comes to verify that all conditions are met), I imagine that it will be even more cumbersome and even more complex to achieve something. So, from a practical perspective and to be effective for the patient, I think that legislation [of CDSUD] is not a good idea’.
Quotation 15: ‘I am not much in favour of legislation of sedation. [. . .] The law will lay down a strict definition and then it will lay down criteria, and then it will lay down etc., etc. And that will be complicated. It is true that we need to reduce the number of abuses that we observe, but we must also not make it [this treatment] inaccessible to people who need it. And I am wary of laws in relation to that [sedation]’.
Quotation 16: ‘I think that everything that is legislated puts a framework so rigid that the people cannot benefit from it because they do not come within the framework or they will benefit too much, because. . . For me, there is a risk of lack of reflection if the law is too rigid. There is also a risk of leaving the most fragile who will not be able to defend themselves or speak. I think there is no need to legislate on that [CDSUD]’.
Quotation 17: ‘I think the Swiss law. . . it defines what is euthanasia. So, it puts a framework on what not to do. Then. . . in my opinion, the most important is to define what is prohibited to do. [. . .] In this way, the legal regulation is well defined in Switzerland, with all the problem of assistance in suicide [. . .]. In any case, I do not feel helpless about this [current Swiss legal regulation]’.

#### Arguments for the legal regulation of CDSUD

Only one argument for the legal regulation of CDSUD was identified. Some participants stated that legislation of CDSUD could reduce the risk of ‘excessive sedation’ (see quotations 1–3).

#### Arguments against the legal regulation of CDSUD

Arguments against the legal regulation of CDSUD were diverse: they focused on medical, epistemological and legal aspects of this practice. Many of the participants based their argument on the risks of the legal regulation of this practice.

##### Medical aspects of CDSUD

Participants pointed out that CDSUD is a therapeutic act in line with the philosophy of palliative care and, for this reason, would not need to be legally regulated in a specific way (see quotations 4–6). Some participants considered clinical practice guidelines on palliative sedation to be sufficient safeguards (see quotations 7 and 8). One participant pointed out that training healthcare professionals to correctly perform palliative sedation would be more important than a law in ensuring safe and correct practice (see quotation 9).

##### Epistemological aspects of CDSUD

One participant suggested that, prior to potential legislation, it would be appropriate to gain more information by conducting research on CDSUD in Switzerland by using the work of the French Society for Support and Palliative Care, for example (see quotation 10).

##### Legal aspects of CDSUD

Several participants stated that any law would be, by definition, imperfect; it would never solve the problem and, despite the law, abuses would always be possible (see quotations 11 and 12). One participant stated that the main problem during the decision-making process with regard to CDSUD is imposing a particular point of view on the patient, and a law would not be able to solve that satisfactorily (see quotation 13). Most of the participants highlighted the potential risks of having detailed legal rules on CDSUD. They stated that it would risk complicating the clinical situation by being too rigid, to the detriment of patients. For example, this could risk delaying the implementation of CDSUD due to additional procedures (see quotation 14). Legislation also risks making this therapy inaccessible for some patients who do not meet the predefined legal criteria, although from a clinical perspective, these patients may need it (see quotation 15). One participant confirmed that legislation of CDSUD may make it impossible for some patients to access the therapy, adding that it may also pass it on to others who, from a clinical perspective, do not need it (see quotation 16). One participant stated that the current Swiss law [Swiss Criminal Code] already regulates all prohibited issues and that seems sufficient (see quotation 17).

### Hypothetical legal regulation of CDSUD in Switzerland, as in France

The question concerning the hypothetical legal regulation of CDSUD in Switzerland, as in France, was put to some of the participants, mainly those who had put forward or tried to put forward arguments for the legislation of this practice. No participant who was asked this question was familiar with the French law on CDSUD. Following the explanation of the French law (see ‘Materials and methods’ section) they agreed that if CDSUD were legally regulated in Switzerland, the rules should in no way be inspired by the French regulation.

## Discussion

Our exploratory study of the opinions of palliative care physicians working in the French-speaking part of Switzerland on the hypothetical legal regulation of CDSUD in their country is the first of its kind. Most of the participants were opposed to having specific legal regulation of CDSUD in Switzerland. Their arguments were diverse: some focused on the medical and epistemological aspects of CDSUD, whereas others emphasized the legal inconvenience of having such regulation. None had the opinion that, if CDSUD were legally regulated in Switzerland, the regulation should be similar to that in France. To the best of our knowledge, no similar studies on this topic have been conducted in Switzerland. Consequently, direct comparisons are not possible.

Contrary to Faeh *et al.*,^18^ our study shows that the participants were not influenced by the French context in terms of wishing to imitate its law. We found that most of them were not even aware of the specific legal regulations regarding CDSUD in France. One of the reasons for this may be that CDSUD is not (yet) broadly practised in France.^27^ From a legal point of view, CDSUD is also legal in Switzerland as long as it acknowledges the general legal conditions for medical interventions (i.e. medical indication and informed consent). The French emphasis on patients’ individual right to request CDSUD is, however, lacking in the Swiss legal system.

Our results could be useful for elaborating a national study aiming to explore this topic in depth. It would be particularly interesting to conduct an interview study with physicians working in the three large linguistic regions of Switzerland: the German-, French- and Italian-speaking areas. Such a study could investigate their opinions on the current situation in Switzerland, its impact on clinical practice in these linguistic regions and whether there is a need to introduce specific legal regulations at the federal or cantonal level (i.e. regional level).

Most of the participants in our study were fully opposed to the legal regulation of CDSUD in Switzerland. They pointed to medical, epistemological and legal aspects of CDSUD. First, they stated that CDSUD is a therapeutic act in line with the philosophy of palliative care and, for this reason, would not need to be legally regulated. On the one hand, this argument may seem intuitive. Indeed, since the beginning of palliative care movement in the 1960s, this treatment of last resort, referred to as ‘sedation’ or ‘terminal sedation’, has been an integral part of the approach.^28,29^ However, from the beginning, this practice has also sparked controversies and debates worldwide.^30,31^

Considered ‘a slow euthanasia’,^30^ ‘an extreme facet of end-of-life sedation’^4^ or ‘an exceptional last resort measure’,^32^ CDSUD can be used inappropriately and contrary to the philosophy of palliative care. International studies have highlighted numerous abuses of this treatment, especially when it is performed as a form of euthanasia – that is, with the intention and the effect to hasten death – in countries with legal regulation of medically assisted dying.^33–35^ Overbeek *et al.*^36^ have even concluded that the legal distinction between euthanasia and ‘palliative or terminal sedation’ may not always be clear in clinical practice. Our previous study has shown that CDSUD is sometimes used as an alternative to assisted suicide in Switzerland.^23^ In this situation, it is legitimate to ask whether a specific regulation of CDSUD may help prevent abuse. This is in line with the opinion of some participants in our study, whereby the legislation of CDSUD could reduce the risk of ‘excessive sedation’. However, due to the lack of studies exploring this topic in depth in France – a country that is unique in its specific legal regulation of CDSUD – it seems difficult to develop this aspect. A recent international survey across eight European countries shows that ‘palliative sedation’ is commonly considered within the general medical law, in legal protections regarding patient autonomy and through clinical practice guidelines.^37^ In some countries, specific recognition of the right to receive ‘palliative sedation’ is included in national laws. However, to the best of our knowledge, studies evaluating the impact of these specific legal regulations on clinical practice, especially on the reduction of the risk of excessive sedation, are lacking thus far. Moreover, it also seems relevant to ask whether such studies would be feasible from a methodological perspective.

Although the Swiss palliative care physicians in our study were rather opposed to a specific law on CDSUD, they acknowledged the importance of some quality assurance and tended to attribute this task to the medical profession. Some study participants considered the present professional guidelines on palliative sedation to be sufficient safeguards. As previously mentioned, in many countries, palliative sedation is only regulated by clinical practice guidelines. Our recent systematic review shows that such documents exist not only in Europe, but also in North America, Australia and Asia.^38^ These texts have been developed to help palliative care physicians address the challenges related to this practice but also to close the gap between research and practice and, eventually, to improve care for patients and their relatives.^39^

However, clinical practice guidelines are not legally binding *per se*, as they are recommendations developed by professional societies, not by a politically legitimate legislative body. In spite of this, they are not legally irrelevant. They can, for instance, be used by courts in assessing physician responsibility by helping to clarify what is an acceptable standard of a particular medical treatment, according to the state of the art.^40^ As some participants of our study mentioned the risk of paternalistic abuse when administering CDSUD, it will be pivotal for clinical practice guidelines to include a statement on ethics, including the principles of respect for patient autonomy and shared decision-making.^41^

It is important to mention that in 2005 a group of experts in the Swiss Society of Palliative Medicine and Care developed clinical practice guidelines on palliative sedation. The document was published in German, French and Italian,^21,42,43^ and interestingly, does not explicitly mention CDSUD. Although this text has existed for more than 15 years, it is still not clear whether and how it is implemented across the country. We know that there are institutional policies in several palliative care units, but the content of these documents is widely ignored; to the best of our knowledge, no review of these texts has been published thus far. Finally, at the time of writing this article, a group of Swiss experts is revising the current clinical practice guidelines.

### Methodological limitations

Our study has one main methodological limitation that should be taken into account when interpreting the results. As previously mentioned, our principal study was focused on CDSUD as an alternative to assisted suicide in Switzerland,^23^ and the present study, as did another one,^24^ was based on a secondary analysis of the data. For the main study,^23^ a progressive inclusion of participants (i.e. inclusion until data saturation was reached) was performed. Data saturation was defined as the point at which no new themes emerged from the analysis of the interviews. The present article shows a sub-analysis of the data from this study as, at the time of the analyses, we discovered interesting elements that were not directly related to the objective of our main study. Data saturation on that particular point of interest in the secondary data analysis could therefore not be sought as the data collection had already finished. Moreover, theoretical saturation was not sought either because there was no literature that would allow us to establish the theoretical saturation point. However, the data were sufficient to explore this topic and to provide ideas for further studies in this area.

## Conclusion

This study, which is the first of its kind in Switzerland, allows a better understanding of why palliative care physicians in the French-speaking part of the country may be reluctant to support the legal regulation of CDSUD. The results of this study could be useful for academic research and for societal debate, although additional studies covering the whole country would be needed to gain a more complete picture of how Swiss palliative care physicians see this question. Further studies are also required to explore the impact of the current lack of legal regulation of CDSUD on clinical practice and on the relationship between physicians, patients and patients’ families.

## Supplemental Material

sj-doc-1-pcr-10.1177_26323524231219509 – Supplemental material for Should continuous deep sedation until death be legally regulated in Switzerland? An exploratory study with palliative care physicians Click here for additional data file. Supplemental material, sj-doc-1-pcr-10.1177_26323524231219509 for Should continuous deep sedation until death be legally regulated in Switzerland? An exploratory study with palliative care physicians by Martyna Tomczyk, Roberto Andorno and Ralf J. Jox in Palliative Care and Social Practice

## Declarations

**Ethics approval and consent to participate:** This study was outside the scope of the Swiss Federal Act on Research involving Human Beings and did not entail any risks. Consequently, the study was deemed exempt from an ethics review by the Swiss Cantonal Ethics Committee. Participation in the study was voluntary and in accordance with ethical guidelines and legal rules. Express oral consent to participation and the interviews being audio recorded were systematically sought and obtained from each participant.

**Consent for publication:** Not applicable.

**Availability of data and materials:** Not all data are available because they contain information that potentially permits the participants to be identified, particularly as the names of the institutions that participated in this study and the characteristics of the participants are explicitly indicated in our previous article.
