# Supplementary material for: Should continuous deep sedation until death be legally regulated in Switzerland? An exploratory study with palliative care physicians
Source: Palliat Care Soc Pract. 2023 Dec 25;17:26323524231219509. doi: 10.1177/26323524231219509 (PMC10752051; doi:10.1177/26323524231219509)
Supplement: sj-doc-1-pcr-10.1177_26323524231219509 – Supplemental material for Should continuous deep sedation until death be legally regulated in Switzerland? An exploratory study with palliative care physicians [file sj-doc-1-pcr-10.1177_26323524231219509.doc]

Should continuous deep sedation until death be legally regulated in Switzerland? An exploratory study with palliative care physicians

**Supplementary File 1: COREQ checklist**

**(COnsolidated criteria for REporting Qualitative research)**

| **Domain 1: Research team and reflexivity** | | |
| --- | --- | --- |
| **Personal characteristics** | | |
| **Interviewer/facilitator** | | Martyna Tomczyk. |
| **Credentials** | | PhD |
| **Occupation** | | Post-doctoral researcher in the ethics of palliative care |
| **Gender** | | Female |
| **Experience and training** | | Background in Medical Humanities (linguistics in respect of the French language, health law, bioethics and medical ethics, with a PhD in medical ethics), nine years of experience in conducting qualitative research. |
| **Relationship with participants** | | |
| **Relationship established** | No relationship (hierarchical, family, etc.) with the participants; in all cases, the interview was the first contact. | |
| **Participant knowledge of the interviewer** | Study carried out as part of a post-doctoral project on the ethics of palliative care, the subject of the study in line with the interviewer’s previous research. | |
| **Interviewer characteristics** | Researcher in palliative care with a background in linguistics, law and ethics, all research in the field of specialized palliative care, nine years of experience in conducting qualitative research. | |
| **Domain 2: Study design** | | |
| **Theoretical framework** | | |
| **Methodological orientation and Theory** | Exploratory qualitative study with deductive thematic analysis. | |
| **Participant selection** | | |
| **Sampling and Method of approach** | The heads of palliative care units with the ‘palliative quality’ label awarded by the Swiss Association for Quality in Palliative Care in the French-speaking part of Switzerland were contacted by email by MT. If the head of a unit agreed to participate in the study, he/she sent an email to all the physicians working in the palliative care unit institution (palliative care unit or associated structures such as a mobile palliative care team) to inform them of the research. Next, physicians who volunteered to participate contacted the researcher and a date was arranged for a meeting. | |
| **Sample size** | Twelve physicians from four palliative care institutions (12 interviews were performed, 10 of which were transcribed and included in the analysis). | |
| **Non-participation** | In total, the researcher contacted nine palliative care units, all of which replied. Six units agreed to participate in the study; four of them had been included when data saturation was reached. Three units declined to participate: two due to work overload and one without giving a reason. | |
| **Setting** | | |
| **Setting of data collection** | Workplace of the participant. | |
| **Presence of non-participants** | Each participant was alone with the researcher. | |
| **Description of sample** | Of the 10 participants whose interviews were included in the study, five were female and five male. The median age was 49 years (38-61). Eight participants were specialized in internal medicine, one in anaesthesiology, and one did not have a medical specialty at the time of this study. Seven participants had training in palliative care and three did not. Seven of the participants had more than 10 years of experience in palliative care, and three participants had less than 10 years. | |
| **Data collection** | | |
| **Interview guide and Repeat interviews** | An interview guide based on a preliminary literature review and containing very general themes as follows:  - General information about the physician (e.g., age, training, experience).  - CDSUD: definition, practice, attitude, law.  - Assisted suicide: definition, attitude, law.  - Assisted suicide request: procedures, difficulties.  - CDSUD and assisted suicide requests: difficult and/or exceptional situations.  These themes were pilot tested with the first three participants and, as no substantive changes were made, their responses were included in the data set. | |
| **Audio/visual recording** | Audio recording. | |
| **Field notes** | No notes. | |
| **Duration** | Total duration of all interviews: 302 minutes (5 hours).  Median duration: 30 minutes.  Shortest and longest durations: 13 minutes and 46 minutes. | |
| **Data saturation** | For the main study [23], the progressive inclusion of participants (i.e., inclusion until data saturation was reached) was performed. Data saturation was defined as the point at which no new themes emerged from the analysis of the interviews. The Comparative Method for Themes Saturation (CoMeTS) was used in order to achieve rigorous data saturation. Moreover, data saturation was in line with theoretical saturation.  The present manuscript shows a sub-analysis of the data from this study; at the time of the analyses, we discovered interesting elements that were not directly related to the objective of our main study. Data saturation could not therefore be sought as the data collection had already finished. Moreover, theoretical saturation was not sought either because there was no literature that would allow us to establish the theoretical saturation point. | |
| **Transcripts returned** | Yes. All transcripts were returned and validated. | |
| **Domain 3: Analysis and findings** | | |
| **Data analysis** | | |
| **Number of data coders** | The data were analysed by MT. RJJ and RA contributed to the process of interpretation for the paper. | |
| **Description of the coding tree** | No. The analysis of the transcripts is not available because they contain information that potentially permits the participants to be identified, particularly as the names of the institutions that participated in this study and the characteristics of the participants are explicitly indicated in the paper related to our principal study. | |
| **Derivation of themes** | Thematic analysis, using a simple framework. Themes identified in advance: Legal regulation of CDSUD in Switzerland (in general), and arguments for and against; and 2) Legal regulation of CDSUD in Switzerland as in France, and arguments for and against. | |
| **Software** | No. Manual analysis. | |
| **Participant checking** | No. | |
| **Reporting** | | |
| **Quotations presented** | All quotations are presented in the Results section of the manuscript. In order to reinforce the anonymity of the participants, the number assigned to each interview during the analysis is not indicated. | |
| **Data and findings consistent** | Considering that the population is homogeneous with regard to profession, and that correlations between age, experience, etc. were not researched, our approach has no impact on the interpretation of the results. | |
| **Clarity of major and minor themes** | The major and minor themes are presented in the Results section and discussed using an interdisciplinary approach in the Discussion section. | |
